# The impact of work pressure on the occupational wellbeing of coaches in grassroots sports schools: a chain mediation effect of emotional intelligence and self-efficacy

**DOI:** 10.3389/fpsyg.2026.1929444

**Published:** 2026-09-22

**Authors:** Duanhua Zhang, Shuyi Zhang, Jianhua Xu

**Affiliations:** Fujian Normal University, Fuzhou, China

**Keywords:** coaches, grass-roots sports schools, intermediary, occupational wellbeing, working pressure

## Abstract

**Objective:**

Occupational wellbeing is an important resource to actively promote the development of social work practice. However, how the working pressure affects the occupational wellbeing of coaches in grass-roots sports schools in China has not been fully discussed. The purpose of this study is to investigate the influence of working pressure on the professional wellbeing of coaches in grass-roots sports schools in China, especially the chain intermediary mechanism of emotional intelligence and self-efficacy related to individual coaches.

**Methods:**

Using cross-sectional design and structured questionnaire, 518 coaches from grass-roots sports schools in China were investigated.

**Results:**

Working pressure can directly and negatively predict the professional wellbeing of coaches in grass-roots sports schools in China, and at the same time, it indirectly shows the relationship with professional wellbeing through three ways. (1) the independent intermediary role of emotional intelligence, (2) the independent intermediary role of self-efficacy, and (3) the chain intermediary role of emotional intelligence and self-efficacy.

**Conclusion:**

These findings illustrate that coaches' emotional intelligence and self-efficacy are positive psychological mechanisms to promote professional wellbeing, and highlight their key roles in improving the professional wellbeing of coaches in grass-roots sports schools in China.

## 1 Introduction

Grassroots sports schools (hereinafter referred to as “sports schools”) are an important part of China's system for identifying, developing, and supplying reserve talent for competitive sports, and coaches constitute the core professional group responsible for youth athlete selection, training and instruction, competition guidance, and developmental management. Compared with many other occupations, the work of sports school coaches is characterized by long training cycles, demanding competition schedules, uncertain performance outcomes, and the coexistence of multiple professional roles, requiring sustained physical and psychological investment. Occupational wellbeing is an important indicator of individuals' positive experiences and psychological functioning in the workplace. It is reflected not only in job satisfaction and positive affect, but also in the sense of value, achievement, competence, belonging, and sustained engagement developed through occupational activities (Ryff, 1989; Van Horn et al., 2004). Previous studies of coaches in other countries have shown that coaches' psychological wellbeing is closely associated with workload, job autonomy, and the availability of work-related resources. For example, research on Australian sports coaches found that workload management and perceived control over coaching responsibilities were significantly associated with psychological wellbeing, while research on Finnish high-performance coaches showed that job demands and job resources were closely related to burnout and work engagement, respectively (Carson et al., 2019; Kaski and Kinnunen, 2021). However, relatively little scholarly attention has been paid to Chinese sports school coaches, a group reported to work more than 320 days per year on average (Yang, 2010), with 25.1% reporting relatively low levels of occupational wellbeing (Han, 2024). Therefore, identifying the key factors associated with occupational wellbeing among sports school coaches is of considerable importance for improving their occupational psychological health, maintaining the stability of the grassroots coaching workforce, and ensuring the sustainable development of reserve talent for competitive sports.

Existing research generally regards working pressure as an important risk factor affecting coaches' occupational psychological states. Compared with specific stressors such as training workload, competition pressure, or role conflict (Thelwell et al., 2008a,b; Olusoga et al., 2009; Didymus et al., 2021), working pressure provides a more comprehensive reflection of coaches' overall stress experiences arising from multiple occupational demands. Accordingly, the present study treats working pressure as the core independent variable for explaining differences in occupational wellbeing among sports school coaches. However, previous studies have focused primarily on the direct relationships between working pressure and outcomes such as burnout, mental health, and work engagement, or have explained these relationships through single factors such as organizational support or coping strategiest (Wright et al., 2023; Kegelaers et al., 2021; Yu and Cheng, 2024). Comparatively less is known about how working pressure is associated with occupational wellbeing through coaches' internal psychological resources, particularly whether multiple psychological resources may operate in a sequential process.

The present study therefore develops a chain mediation model, drawing on the Job Demands-Resources theory and Conservation of Resources theory as its basic theoretical foundations. Both perspectives emphasize that persistent job demands may deplete individuals' psychological resources, whereas the personal resources they possess and are able to mobilize can help them cope with stress and maintain positive occupational functioning (Hobfoll, 1989; Bakker and Demerouti, 2017). We propose that emotional intelligence and self-efficacy may serve as two sequential psychological mediators between working pressure and occupational wellbeing. Emotional intelligence refers to the ability to perceive, understand, use, and regulate one's own and others' emotions, and is particularly relevant in coaching contexts in which individuals must continuously manage competition outcomes, athletes' emotional states, interpersonal interactions, and organizational demands. Research has shown that higher emotional intelligence can help individuals reduce working pressure and its adverse psychological consequences (Ogińska-Bulik, 2005), while studies among sports coaches have also demonstrated that emotion regulation is closely related to coaching efficacy beliefs (Teques et al., 2019a,b). Self-efficacy refers to individuals' beliefs in their ability to cope effectively with difficulties and accomplish work-related tasks. For sports school coaches, athlete development occurs over a relatively long period, while training outcomes and competitive performance are often uncertain. Thus, maintaining confidence in one's coaching competence despite persistent stress and limited short-term feedback may be particularly important for sustaining positive occupational experiences. On this basis, the present study proposes a chain mediation pathway of “working pressure → emotional intelligence → self-efficacy → occupational wellbeing.”

It should be noted that the present study adopts a cross-sectional design and therefore aims to examine patterns of association and potential mediating pathways among working pressure, emotional intelligence, self-efficacy, and occupational wellbeing rather than to establish strict causal relationships. Specifically, this study seeks to examine the relationship between working pressure and occupational wellbeing among sports school coaches and to test the potential independent and sequential mediating roles of emotional intelligence and self-efficacy. Theoretically, this study extends the focus of previous research from negative occupational outcomes, such as working pressure and burnout, to occupational wellbeing as a positive psychological outcome and further explores the sequential roles of two internal psychological resources, emotional intelligence and self-efficacy, thereby deepening the understanding of the psychological mechanisms associated with coaches' occupational wellbeing under highly demanding working conditions. Practically, if the proposed model is supported, the findings may provide a more differentiated basis for interventions aimed at reducing excessive work pressure, strengthening emotional recognition and regulation, and enhancing occupational self-efficacy, while also offering preliminary evidence for future longitudinal and experimental studies seeking to verify the temporal ordering and causal relationships among these variables.

## 2 Literature review and hypothesis development

### 2.1 Work pressure and occupational happiness

In the field of occupational psychology, the Job Demands-Resources (JD-R) model has been widely used to explain the relationship between work pressure and psychological health. The JD-R model suggests that individuals are more likely to maintain high levels of work engagement and wellbeing when job demands and job resources are relatively balanced, whereas excessive demands combined with insufficient resources may lead to persistent work pressure (Schaufeli and Taris, 2013). For coaches in grassroots sports schools, intensive training schedules, frequent competitions, and organizational responsibilities may place substantial demands on their available resources. Such an imbalance can contribute to resource depletion, accumulated psychological stress, and emotional exhaustion, thereby reducing occupational wellbeing. Previous studies have shown that coaches' work pressure may stem from factors such as responsibility for competitive performance, organizational expectations, time constraints, and role conflict, all of which may negatively affect their subjective wellbeing (Potts et al., 2023). Given the distinctive nature of the coaching profession, psychological stress may become particularly pronounced when job demands continuously exceed available resources (Olusoga et al., 2009). For example, research has found that burnout symptoms among coaches under high job demands are closely associated with an imbalance between demands, effort, and rewards, while also highlighting the important role of the school work environment in the development of coaches' stress and burnout (Yan et al., 2023). Moreover, when coaches perceive that their abilities and available resources are insufficient to meet existing work demands, their perceived work pressure may be further intensified, and this appraisal process itself may adversely affect their subjective wellbeing. When individuals perceive that they lack sufficient resources to cope with stressors, they are more likely to experience helplessness and frustration, which may not only impair psychological health but also reduce occupational wellbeing and life satisfaction (Halbesleben et al., 2014). Therefore, coaches in grassroots sports schools who are exposed to persistently high job demands may face not only substantial training and managerial pressures, but also an elevated risk of reduced occupational wellbeing due to insufficient organizational support, limited time resources, and restricted opportunities for professional development. Although a considerable body of research has examined work pressure and its psychological consequences among sports coaches, systematic research on specific psychological protective factors for grassroots sports school coaches under high-stress working conditions remains limited.

Based on the above analysis, this study puts forward hypothesis H1: working pressure has a direct effect on the occupational wellbeing of sports school coaches.

### 2.2 Work pressure, emotional intelligence and occupational wellbeing

Emotional intelligence refers to an individual's ability to perceive, understand, use, and regulate emotions in oneself and others. It encompasses not only the perception and evaluation of emotional information but also the process of managing emotional responses through appropriate strategies. This ability is particularly important for coaches in maintaining psychological adjustment within complex occupational environments (Chan and Mallett, 2011). In the coaching context, tasks such as training program design, competition guidance, interpersonal interaction, and team management are often accompanied by intense emotional fluctuations and stress experiences. Higher emotional intelligence can help coaches regulate their emotional responses more effectively, thereby reducing stress-induced negative experiences and improving psychological adjustment. Suppose the model is as shown in Figure 1. Previous studies have shown that emotional intelligence among sports coaches is significantly associated with psychological wellbeing, job satisfaction, and emotional exhaustion. Coaches with higher emotional intelligence are also more likely to adopt constructive emotion-regulation strategies, enabling them to maintain better psychological health under stressful conditions (Zajonz et al., 2024). In addition to its direct influence on emotion regulation and psychological experiences, emotional intelligence may indirectly enhance occupational wellbeing by strengthening coaches' ability to perceive and utilize external resources. For example, coaches with higher emotional intelligence may be better able to derive positive feedback from training evaluations, fluctuations in athlete performance, or organizational assessments and transform such feedback into psychological resources that further promote wellbeing (Watson and Kleinert, 2019).

**Figure 1:**
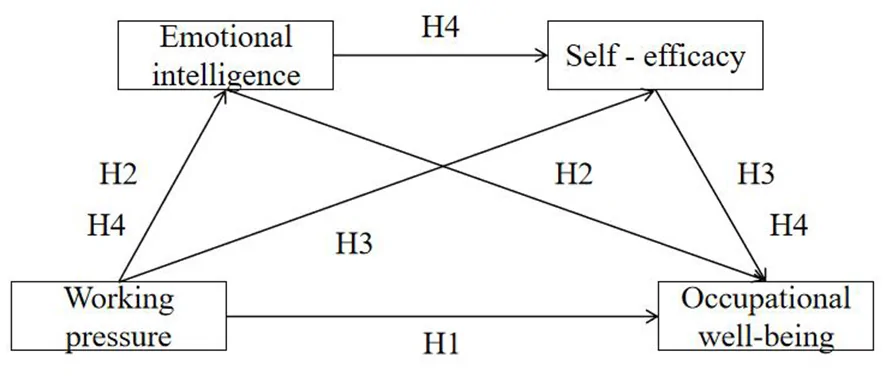
Hypothesized model of the study.

Moreover, higher emotional intelligence may buffer the negative effects of stress by improving the quality of coping strategies. Previous research has indicated that coaches with higher levels of emotional intelligence and intrinsic motivation are more likely to employ constructive coping strategies, such as problem solving and cognitive reappraisal. These strategies can help reduce negative emotions and burnout associated with stressful experiences and, in turn, enhance occupational wellbeing (Grijalbo et al., 2022). Similarly, when coaches experience negative emotions, the interaction between constructive coping strategies and emotional intelligence may contribute to better psychological health and higher levels of wellbeing, further highlighting the important role of emotional intelligence in the relationship between stress and wellbeing (Lee and Cho, 2023). Therefore, for coaches in grassroots sports schools, emotional intelligence may function as an important psychological resource that helps them regulate emotional responses, maintain positive emotional experiences and psychological balance, strengthen their perceived control over stressful situations, adopt more adaptive coping strategies, and make more effective use of external resources. Through these processes, emotional intelligence may serve as an important mechanism linking work pressure to occupational wellbeing and provide psychological support for maintaining or improving occupational wellbeing under conditions of prolonged work pressure.

Based on the above analysis, this study proposes Hypothesis H2: Emotional intelligence mediates the relationship between working pressure and the occupational wellbeing of sports school coaches.

### 2.3 Work pressure, self-efficacy and career happiness

Self-efficacy is also an important psychological resource that influences the occupational wellbeing of grassroots sports school coaches. It refers to an individual's belief in their ability to successfully accomplish specific tasks and achieve desired outcomes (Bandura, 1977). In the field of coaching science, self-efficacy is often conceptualized as coaching efficacy, which reflects the degree to which coaches believe they can influence athletes' learning, training performance, and developmental outcomes. It typically includes dimensions such as game strategy, motivation, instruction, and character building (Feltz et al., 1999). For grassroots sports school coaches, high self-efficacy means that they remain confident in their ability to solve problems and facilitate athlete development even when facing training difficulties, performance fluctuations, athlete management challenges, and resource constraints. For example, research has shown that coaching efficacy can predict coaches' leadership styles (Sullivan and Kent, 2003) and is associated with coaching behaviors, athlete satisfaction, and team performance (Myers N. D. et al., 2005; Myers S. et al., 2005). Therefore, when coaches have high confidence in their ability to cope with challenges, they are more likely to adopt constructive strategies to manage stress, enhance work engagement and job satisfaction, and strengthen occupational wellbeing (Elmas et al., 2026). However, sustained high working pressure may undermine this belief. When grassroots coaches are confronted with persistent challenges such as limited training effectiveness, unstable performance outcomes, fluctuations in athlete condition, insufficient organizational support, or overly simplistic evaluation systems, they may begin to doubt their coaching competence. This, in turn, reduces their self-efficacy, increases psychological burden under stressful conditions, and limits their ability to experience a sense of control and positive engagement in work, thereby diminishing occupational wellbeing (Stebbings et al., 2011). Related research also indicates that satisfaction of coaches' psychological needs and their self-efficacy are key determinants of psychological health and wellbeing. As core psychological resources, they help coaches better manage stress and maintain a positive occupational state (Norris et al., 2017). Therefore, self-efficacy may play an important mediating role in the relationship between working pressure and the occupational wellbeing of grassroots sports school coaches.

Based on the above analysis, this study puts forward the hypothesis H3: Self-efficacy mediates the relationship between working pressure and the occupational wellbeing of sports school coaches.

### 2.4 Work pressure, emotional intelligence, self-efficacy and occupational wellbeing

The Conservation of Resources (COR) theory proposes that individuals can resist working pressure and maintain or enhance occupational wellbeing by accumulating and utilizing valuable psychological resources (Hobfoll, 1989). According to this theory, the accumulation and conservation of psychological resources not only help coaches obtain more positive work experiences but also effectively reduce the negative impact of working pressure (Gould et al., 2002). Among these psychological resources, emotional intelligence and self-efficacy are particularly critical. Emotional intelligence enables coaches to perceive, understand, and regulate their own emotions as well as those of others, thereby allowing them to better cope with emotional challenges in the face of complex training tasks and high-intensity competitive pressures, and to alleviate negative emotions arising from stress (Laborde et al., 2016). Empirical studies indicate that in coaching contexts characterized by high emotional demands, coaches' emotional intelligence can mitigate exhaustion caused by emotional labor, thereby helping to maintain psychological health and enhance levels of wellbeing (Lee and Chelladurai, 2016). Self-efficacy, in turn, enables coaches to maintain confidence and adopt positive coping strategies when facing training difficulties, fluctuations in athlete performance, or organizational demands, thereby reducing the negative impact of stress and improving occupational wellbeing (Bandura, 1997). Research has shown that coaches with higher levels of self-efficacy experience lower emotional exhaustion and significantly higher job satisfaction and psychological wellbeing under high job-demand conditions (Moritz et al., 2000). In addition, emotional intelligence and self-efficacy may form a sequential mechanism that jointly buffers the negative effects of working pressure on wellbeing (Fletcher and Hanton, 2003). Specifically, coaches with high emotional intelligence are better able to regulate emotions and avoid interpreting temporary setbacks as a lack of personal ability, thereby strengthening their self-efficacy and further maintaining occupational wellbeing (Henriksen and Stambulova, 2023).

Bandura's social cognitive theory further posits that self-awareness, self-regulation, and emotional control are crucial for the development of self-efficacy. Such beliefs about one's capabilities not only influence how individuals set goals and confront challenges but also determine how they cope with problems in stressful environments (Bandura, 1991). For coaches, emotional intelligence as the ability to perceive, understand, and regulate emotions can significantly influence the formation and development of self-efficacy. On the one hand, coaches with higher emotional intelligence are better able to identify and manage stress experiences that arise during training and competition. This emotional sensitivity and regulation capacity can enhance their sense of control and foster a more positive attitude toward task challenges (Teques et al., 2019a,b). On the other hand, higher emotional intelligence enables coaches to better understand and respond to the emotional reactions of athletes, colleagues, and others, thereby establishing positive emotional feedback loops in interactions and strengthening both team cohesion and self-efficacy (Hwang et al., 2013). Accordingly, coaches with higher emotional intelligence are more capable of managing emotions proactively, setting realistic expectations, and flexibly adjusting strategies to meet the psychological needs of athletes and teams. These experiences enhance their sense of control and achievement in training tasks, which in turn further reinforces their self-efficacy beliefs (Thelwell et al., 2008a,b). Therefore, emotional intelligence and self-efficacy are likely to form a chain mediation mechanism between working pressure and occupational wellbeing among sports school coaches. Specifically, high emotional intelligence may enhance coaches' self-efficacy, thereby buffering the negative impact of working pressure on wellbeing and jointly promoting occupational wellbeing under high-pressure working conditions.

Based on the above analysis, this study puts forward the hypothesis H4: Emotional intelligence and self-efficacy form a chain mediating effect in the relationship between working pressure and the occupational wellbeing of sports school coaches.

## 3 Methodology

### 3.1 Research design and sampling method

During the study design stage, the minimum required sample size was estimated with reference to effect sizes reported in relevant studies, while also taking into account the methodological characteristics of previous similar research and their differences from the present study. Based on these considerations, at least 300 participants were considered necessary to ensure adequate statistical power (Villafaina et al., 2021). Accordingly, an electronic questionnaire was developed using the Wenjuanxing online survey platform. Data collection began in December 2025, with the initial distribution of questionnaires to coaches in Fujian and Zhejiang Provinces, China. In April 2026, taking advantage of work-related internship opportunities, the sample was further expanded to grassroots sports school coaches across different provinces and municipalities in China through snowball sampling based on personal referrals. A total of 600 questionnaires were distributed (Table 1). After excluding questionnaires containing errors or irregular responses, 518 valid questionnaires were retained, yielding an effective response rate of 86.33%. Among the participating coaches, 329 were male (63.51%) and 189 were female (36.49%). To enhance the representativeness of the sample and the authenticity of the data, efforts were made to recruit coaches with different levels of coaching experience. Specifically, 93 coaches (17.95%) had 5 years or less of coaching experience, 168 (32.43%) had 6–10 years of experience, 149 (28.76%) had 11–15 years of experience, and 108 (20.85%) had more than 15 years of experience, thereby ensuring diversity in coaching tenure. In addition, the participants were drawn from the major geographical regions of China, including North China, South China, East China, Central China, Southwest China, Northwest China, and Northeast China. However, due to limitations in research resources and practical conditions, the present study did not include coaches from Taiwan Province or the Hong Kong and Macao Special Administrative Regions of China.

**Table 1:** Demographic characteristics of the participants (*N* = 518).

Characteristics	Frequency (*n*)	Percentage (%)
Gender		
Male	329	63.51
Female	189	36.49
Teaching years		
Less than 5 years	93	17.95
6–10 years	168	32.43
11–15 years	149	28.76
More than 15 years	108	20.85
Academic degree		
Junior college and below	95	18.34
Undergraduate course	294	56.76
Master degree or above	129	24.90
Marital status		
Married	332	64.09
Unmarried	146	28.19
Others	40	7.72
Age		
25 years old and below	42	8.11
26–30 years old	124	23.94
31–35 years old	189	36.49
36–40 years old	95	18.34
40 years old and above	68	13.13
Location		
North China	76	14.67
Central China	58	11.20
East China	93	17.95
South China	88	16.99
Southwest China	93	17.95
Northwest China	58	11.20
Northeast China	52	10.04

North China includes(Beijing, Tianjin, Hebei, Shanxi and Inner Mongolia Autonomous Region); Central China includes(Henan Province, Hubei Province and Hunan Province); East China includes(Shanghai, Jiangsu, Zhejiang, Anhui, Fujian, Jiangxi, Shandong); South China includes(Guangdong Province, Guangxi Zhuang Autonomous Region, Hainan Province,); Southwest China includes(Chongqing, Sichuan, Guizhou, Yunnan and Xizang Autonomous Region); Northwest China includes(Shaanxi Province, Gansu Province, Qinghai Province, Ningxia Hui Autonomous Region and Xinjiang Uygur Autonomous Region); Northeast China includes(Heilongjiang Province, Jilin Province and Liaoning Province).

### 3.2 Data collection procedure

The research procedure was strictly designed. Prior to data collection, the research team clearly explained the purpose and content of the study to participating coaches and provided informed consent forms to obtain their willingness to participate. Ultimately, all participating sports school coaches signed informed consent forms, ensuring that the survey was conducted on a voluntary and informed basis. In addition, this study strictly adhered to international ethical standards, including the ethical guidelines of the American Psychological Association (APA). No material incentives were provided to participants, and strict confidentiality was maintained regarding participants' personal information and questionnaire data. The processes of data collection and processing were jointly completed with graduate students specializing in sports psychology, all of whom received professional training to ensure the accuracy of questionnaire administration, data organization, and data entry. During the questionnaire completion process, the research team provided detailed instructions regarding the survey procedure and the scope of data usage, clearly informing participants that all information would be used solely for research purposes and would be handled anonymously. Participants were required to complete the questionnaire independently.

### 3.3 Data analysis

Guided by the hypotheses proposed in advance, this study systematically processed and analyzed the collected data. First, the questionnaire data were screened, and invalid or incomplete responses were excluded to ensure the reliability of the subsequent analyses. Second, descriptive statistics and correlation analyses were conducted for the main study variables. The means, standard deviations, and Pearson correlation coefficients of working pressure, emotional intelligence, self-efficacy, and occupational wellbeing were calculated to examine their basic distributional characteristics and bivariate associations. Finally, the PROCESS macro (Version 4.0) in SPSS 25.0 was used to test the chain mediation effect. Work pressure was specified as the independent variable, occupational wellbeing as the dependent variable, and emotional intelligence and self-efficacy were entered sequentially as mediators, with gender, years of coaching experience, and educational level included as covariates. The significance of the indirect effects was assessed using a bias-corrected bootstrap procedure with 5,000 resamples and 95% confidence intervals. An indirect effect was considered statistically significant when the corresponding confidence interval did not include zero.

### 3.4 Measurement instruments

#### 3.4.1 Working pressure scale

This study draws lessons from the work pressure Scale compiled by Wang (2025) on the basis of referring to Kahn and Byosiere (1992) and Sonnentag and Fritz (2015). Compared with other scales for measuring work pressure, this scale has more dimensions and a wider understanding, which is helpful for in-depth investigation of the work pressure levels of different coaches, thus providing more powerful support for the development of this study. The purpose of this scale is to measure work pressure from the perspective of coaches. There are 18 items in this scale (for example, “My work task is heavy and complicated” and “I often feel that my ability is insufficient at work”). According to Wang Yanru's classification of work pressure, this study divides all the items into five dimensions: workload, work environment, personal skill matching, salary and work-family conflict, and assigns them with a five-point Likert scale (1 = very inconsistent, 5 = very consistent). The higher the score, the greater the work pressure of coaches. The verification results of the measurement model are fitted as *X*^2^/ df = 0.875, goodness of fit index (GFI) = 0.997, comparative fitting index (CFI) = 1.000, Tucker-Lewis index (TLI) = 1.000, approximate root mean square error (RMSEA) = 0.025, and standardized root mean square residual (SRMR) = The Cronbach's α coefficient of the total table of this study is 0.913, and the Cronbach's α coefficients of each coach's work pressure dimension are 0.893, 0.869, 0.664, 0.717 and 0.751, respectively.

#### 3.4.2 Coach occupational wellbeing scale

In this study, referring to Zhao Bin's “Questionnaire of occupational wellbeing for Primary and Secondary School Teachers” (Zhao et al., 2012), combined with the professional characteristics of coaches in sports schools, the scale was adjusted adaptively to reflect the occupational wellbeing level of grassroots coaches more accurately. The revised scale includes six dimensions, with a total of 22 items (for example, “I often study in monologs or discuss coaching with colleagues” and “sports teams attach importance to improving the working environment of coaches”), among which “I often get tired of my job” is a reverse scoring item. The questionnaire adopts five-point Likert scale scoring method, and the higher the score, the stronger the occupational wellbeing of coaches. In this research sample, the Cronbach's α coefficient of the total table is 0.936, and the Cronbach's α coefficients of each dimension are 0.834, 0.833, 0.915, 0.934, 0.916 and 0.914, which all reach a good level, indicating that the scale is reliable in internal consistency and can effectively measure the occupational wellbeing of sports school coaches. The validation results of the measurement model are *X*^2^/ df = 0.018, goodness of fit index (GFI) = 1.000, comparative fitting index (CFI) = 1.000, Tucker-Lewis index (TLI) = 1.000, and approximate root mean square error (RMSEA) = 0.000, which shows that the scale has excellent structural validity.

#### 3.4.3 Emotional intelligence scale

The emotional intelligence scale used in this study was compiled by K. Wong and revised by Wang (2010). The scale includes four dimensions: self-emotional awareness, others' emotional awareness, emotional management and emotional use, with a total of 16 items (for example, “I usually know the reasons why I feel certain things” and “I have strong control over my emotions”). The scale adopts five-point Likert scoring method, and the higher the score, the higher the individual's emotional intelligence level. In this research sample, the validation results of the measurement model are fitted as *X*^2^/ df = 1.106, goodness of fit index (GFI) = 0.994, comparative fitting index (CFI) = 0.999, Tucker-Lewis index (TLI) = 0.999, and approximate root mean square error (RMSEA) = 0.014, all of which are acceptable. The Cronbach's α coefficient of the total table in this study is 0.924, and the Cronbach's α coefficients of each dimension are 0.942, 0.943, 0.936 and 0.935, respectively.

#### 3.4.4 Self-efficacy scale

The self-efficacy scale used in this study was compiled by Professor R. Schwarzer and translated and revised by scholars such as Zhang Jianxin (Wang et al., 2001). The scale initially contained 20 items (for example, “If I try my best, I can always solve the training problem” and “Even if others object to me, I can still get the training effect I want”). Later, it was simplified and revised to 10 items, and a five-point Likert scoring method was adopted. The higher the score, the higher the level of individual self-efficacy. In this research sample, the verification results of the measurement model are fitted as *X*^2^/ df = 0.912, goodness of fit index (GFI) = 0.988, comparative fitting index (CFI) = 1.000, Tucker-Lewis index (TLI) = 1.000, and approximate root mean square error (RMSEA) = 0.000, all of which are in line with the requirements. It shows that the scale has high measurement reliability in terms of structural validity and can be effectively used to evaluate the self-efficacy level of coaches in sports schools. The Cronbach's α coefficient of the scale is 0.977.

## 4 Results

### 4.1 Common method bias test

Given that the questionnaire in this study was completed anonymously and that some items relied on respondents' subjective judgments, there may be a risk of common method bias. To address this issue, Harman's single-factor test was conducted through an unrotated exploratory factor analysis of all 66 measurement items. The results showed that 16 factors with eigenvalues greater than 1 were extracted, and the first factor accounted for 28.024% of the total variance, which is below the critical threshold of 40%. Therefore, this study concludes that no significant common method bias exists among the variables, providing a reliable foundation for subsequent data analysis and hypothesis testing.

### 4.2 Descriptive statistics and correlation analysis

Independent samples *t*-test results indicated that there were significant gender differences among sports school coaches in working pressure (*t* = 4.546, *p* < 0.001), occupational wellbeing (*t* = 3.691, *p* < 0.001), emotional intelligence (*t* = 2.059, *p* < 0.05), and self-efficacy (*t* = 2.597, *p* < 0.01). Moreover, male coaches scored significantly higher than female coaches across all four variables. One-way analysis of variance (ANOVA) results showed that coaches with different lengths of coaching experience also exhibited significant differences in working pressure (*F* = 12.048, *p* < 0.001), occupational wellbeing (*F* = 10.621, *p* < 0.001), emotional intelligence (*F* = 10.246, *p* < 0.001), and self-efficacy (*F* = 5.172, *p* < 0.01).

Partial correlation analysis was conducted to examine the relationships among working pressure, emotional intelligence, self-efficacy, and occupational wellbeing of sports school coaches after controlling for gender and years of coaching experience(Table 2). The results showed that working pressure was significantly negatively correlated with emotional intelligence (*r* = −0.439, *p* < 0.001), self-efficacy (*r* = −0.422, *p* < 0.001), and occupational wellbeing (*r* = −0.481, *p* < 0.001). Emotional intelligence was significantly positively correlated with self-efficacy (*r* = 0.360, *p* < 0.001) and occupational wellbeing (*r* = 0.527, *p* < 0.001). In addition, self-efficacy was also significantly positively correlated with occupational wellbeing (*r* = 0.430, *p* < 0.001). All correlation coefficients reached statistical significance, indicating that the relationships among the variables are stable and not substantially influenced by gender or age.

**Table 2:** Correlation analysis of study variables (*N* = 518).

	M ±SD	Working pressure	Emotional intelligence	Self-efficacy	Occupational wellbeing
Working pressure	2.89 ± 0.82	1			
Emotional intelligence	2.90 ± 0.78	−0.439^***^	1		
Self-efficacy	2.90 ± 0.88	−0.422^***^	0.360^***^	1	
Occupational wellbeing	2.91 ± 1.17	−0.481^***^	0.527^***^	0.430^***^	1

^**^p < 0.01, ^***^p < 0.001; the same applies hereinafter.

### 4.3 Multicollinearity test

Given the significant correlations among the variables, there was a potential risk of multicollinearity. Therefore, a collinearity diagnostic was conducted with occupational wellbeing as the dependent variable and working pressure, emotional intelligence, and self-efficacy as independent variables, and all predictor variables were standardized prior to analysis. The results showed that the tolerance values of the predictor variables were 0.842, 0.802, and 0.802, respectively, all exceeding the threshold of 0.10. The corresponding variance inflation factor (VIF) values were 1.188, 1.247, and 1.246, respectively, all below 5. These findings indicate that no multicollinearity problem exists among the variables, and thus the data are suitable for subsequent chain mediation analysis.

### 4.4 Mediation model test

With working pressure as the independent variable, emotional intelligence and self-efficacy as mediating variables, and occupational wellbeing of sports school coaches as the dependent variable, the PROCESS macro (Model 6) developed by Hayes (2017) in SPSS (Version 3.5) was used to conduct a bootstrap mediation analysis. A total of 5,000 bootstrap resamples were generated, and a 95% confidence interval was set as the default parameter for significance testing.

After controlling six demographic variables, such as gender, teaching years, education background, marital status, age and work area, this paper takes emotional intelligence and self-efficacy as mediating variables, work pressure as independent variable and occupational wellbeing as dependent variable, and uses a simple mediating model to test whether emotional intelligence and self-efficacy play an mediating role between work pressure and occupational wellbeing, respectively. As the results in Table 3 and Figure 2 show, firstly, work pressure not only directly and significantly negatively predicts occupational happiness (β = −0.281, *P* < 0.001), but also significantly negatively predicts emotional intelligence (β = −0.426, *P* < 0.001) and self-efficacy (β = −0.267, *P* < 0.001). Secondly, emotional intelligence significantly positively predicted self-efficacy (β = 0.183, *P* < 0.001) and occupational wellbeing (β = 0.429, *P* < 0.001); Thirdly, self-efficacy significantly positively predicted occupational wellbeing (β = 0.294, *P* < 0.001), and Bootstrap test showed that 95% confidence intervals of the three mediating paths did not include 0, indicating that emotional intelligence and self-efficacy played a complete chain mediating effect between work pressure and occupational wellbeing.

**Table 3:** Regression analysis of the chain mediation model of working pressure, emotional intelligence, and self-efficacy on occupational wellbeing of sports school coaches.

Regression equation		Global fitting index			Coefficient of regression				
Outcome variable	Predictor variable	*R*	*R* ^2^	*F*	β	SE	*t*	LLCI	ULCI
Emotional intelligence	Working pressure	0.493	0.243	23.4331^***^	−0.426	0.0385	−11.067^***^	−0.502	−0.351
	Gender				−4.704	1.1392	−4.1295^***^	−6.943	−2.466
	Teaching years				4.718	0.5526	8.5375^***^	3.632	5.804
	Academic degree				0.284	0.8236	0.3451	−1.334	1.902
	Marital status				0.570	0.8592	0.6628	−1.119	2.258
	Age				−0.099	0.4796	−0.2076	−1.042	0.843
	Working area				0.1264	0.2898	0.4363	−0.4429	0.6957
Self-efficacy	Working pressure	0.498	0.248	20.952^***^	−0.267	0.0358	−7.460^***^	−0.337	−0.197
	Emotional intelligence				0.183	0.0370	4.956^***^	0.111	0.256
	Gender				−3.648	0.9676	−3.7706^**^	−5.549	−1.747
	Teaching years				2.269	0.4936	4.5982^***^	1.300	3.239
	Academic degree				0.039	0.6882	0.0571	−1.313	1.391
	Marital status				−0.129	0.7182	−0.1797	−1.540	1.282
	Age				0.104	0.4007	0.2593	−0.683	0.891
	Working area				0.0989	0.2422	0.408	−0.377	0.575
Occupational wellbeing	Working pressure	0.667	0.445	45.299^***^	−0.281	0.0479	−5.881^***^	−0.375	−0187
	Emotional intelligence				0.429	0.0480	8.937^***^	0.335	0.524
	Self-efficacy				0.294	0.0562	5.231^***^	0.184	0.404
	Gender				−0.282	0.0479	−5.8919^***^	−7.683	−2.793
	Teaching years				0.429	0.0480	8.9206^***^	1.518	4.029
	Academic degree				0.293	0.0562	5.2134	−3.851	−0.420
	Marital status				−5.238	1.2445	−4.2091	−3.119	0.461
	Age				2.773	0.6391	4.3398	−1.469	0.529
	Working area				−2.136	0.8730	−2.4461	−0.348	0.859
Occupational wellbeing	Working pressure	0.548	0.300	31.263^***^	−0.566	0.0457	−12.366^***^	−0.656	−0.476
	Gender				−8.576	1.3534	−6.3368^***^	−11.235	−5.917
	Teaching years				5.714	0.6565	8.7031^***^	4.424	7.004
	Academic degree				−1.987	0.9785	−2.0307	−3.909	−0.065
	Marital status				−1.092	1.0208	−1.0698	−3.098	0.913
	Age				−0.488	0.5697	−0.856	−1.607	0.632
	Working area				0.3454	0.3443	1.0034	−0.331	1.022

^**^p < 0.01, ^***^p < 0.001; the same applies hereinafter.

**Figure 2:**
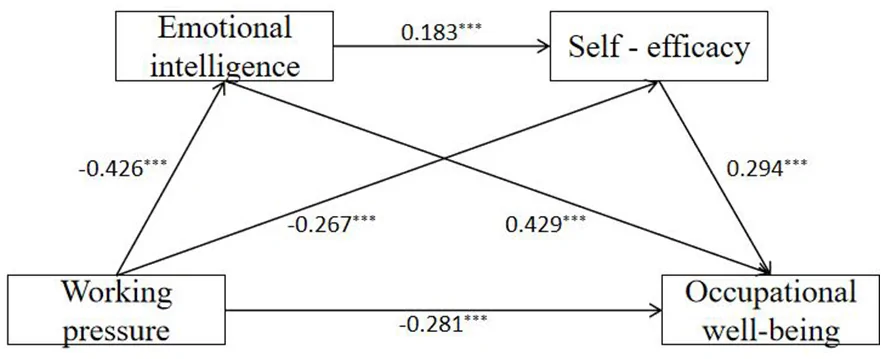
Chain mediation model of emotional intelligence and self-efficacy. ^***^p < 0.001.

After controlling six demographic variables, such as gender, teaching years, education background, marital status, age and work area, this paper takes emotional intelligence and self-efficacy as mediating variables, work pressure as independent variable and occupational wellbeing as dependent variable, and uses a simple mediating model to test whether emotional intelligence and self-efficacy play an mediating role between work pressure and occupational wellbeing, respectively. As shown in Table 4, under the condition of controlling the five variables of gender, teaching years, education background, marital status and work area, emotional intelligence and self-efficacy are used as mediating variables, and the chain mediation model is used to test the chain mediation role of them in work pressure and occupational wellbeing. First of all, through intermediary analysis, it is concluded that the total intermediary effect is −0.2838, accounting for 50.15% of the total effect, and the 95% confidence interval does not include 0, indicating that the model with emotional intelligence and self-efficacy as chain intermediary variables is established and has good reliability. Secondly, emotional intelligence and self-efficacy are brought into the relationship model between work pressure and

**Table 4:** Test of the chain mediation effects of emotional intelligence and self-efficacy in the relationship between working pressure and occupational wellbeing.

Mediating effect path	Standardized indirect effect value	Boot SE	95% confidence interval	Relative effect
Total effect	−0.5659	0.0457	[−0.6558, −0.4760]	100%
Direct effect	−0.2821	0.0479	[−0.3761, −0.1880]	49.85%
Working pressure → emotional intelligence → self-efficacy → occupational wellbeing	−0.0228	0.0069	[−0.0379, −0.0108]	4.03%
Working pressure → emotional intelligence → occupational wellbeing	−0.1827	0.0248	[−0.2326, −0.1354]	32.28%
Working pressure → self-efficacy → occupational wellbeing	−0.0783	0.0184	[−0.1163, −0.0445]	13.84%
Total intermediary effect	−0.2838	0.0316	[−0.3487, −0.2252]	50.15%

occupational wellbeing, and there are four paths for work pressure to affect occupational wellbeing. The direct effect of Path 1 (work pressure → occupational wellbeing) is −0.2821, accounting for 49.85%. Path 2 (work pressure → emotional intelligence → self-efficacy → occupational wellbeing) has a chain mediating effect of −0.0228, accounting for 4.03%; Path 3 (work pressure → emotional intelligence → occupational wellbeing) has a single mediating effect of −0.1827, accounting for 32.28%; Path 4 (work pressure → self-efficacy → occupational wellbeing) has a single mediating effect of −0.0783, accounting for 13.84%. None of the 95% confidence intervals of the above four paths contains 0, which shows that in the correlation between work pressure and coaches' professional wellbeing, work pressure can directly affect their professional wellbeing, emotional intelligence and self-efficacy can also independently mediate, and they can also play a chain mediation effect together, providing effective empirical support for the relevant assumptions of this study.

## 5 Discussion

### 5.1 The effect of working pressure on the occupational wellbeing of sports school coaches

The present study found a significant negative relationship between work pressure and occupational wellbeing among sports school coaches, thereby supporting Hypothesis 1. This finding is consistent with previous research (Wright et al., 2023; Upenieks et al., 2023), suggesting a relatively stable association between higher levels of work pressure and lower levels of positive occupational experience. However, this result should not be interpreted simply as indicating that “the heavier the workload, the lower the occupational wellbeing.” According to the Job Demands-Resources theory, whether job demands lead to adverse outcomes depends largely on how individuals appraise these demands and whether they possess sufficient resources to cope with them. In particular, when job demands are perceived as uncontrollable, insufficiently supported, or obstructive to goal attainment, they are more likely to become hindrance demands and continuously deplete psychological resources (Crawford et al., 2010). Previous research has also shown that coaches' ability to manage workload and their perceived control over work are closely related to psychological wellbeing, with lower levels of job autonomy and perceived control being associated with poorer psychological states (Carson et al., 2019). Therefore, for sports school coaches, intensive training schedules, frequent competitions, athlete management, and administrative responsibilities may not necessarily reduce occupational wellbeing in themselves; rather, the more detrimental condition may arise when high demands coexist with low control, insufficient support, and inadequate rewards, resulting in persistent resource imbalance. In grassroots sports school settings, coaches are often held directly accountable for outcomes such as athletes' competitive performance, the development and progression of reserve talent, and team management, yet these outcomes are simultaneously influenced by multiple factors, including athletes' innate potential, injuries, training conditions, competitive environments, and policy resources, and therefore cannot be determined solely by coaches' individual efforts (Pilkington et al., 2022; Kegelaers et al., 2021). This may create a “high responsibility-limited control” occupational situation in which coaches are expected to take responsibility for outcomes over which they do not have complete control. Moreover, when evaluation systems place greater emphasis on competitive results, while training processes, educational contributions, and long-term development are not readily translated into professional recognition, coaches' sustained efforts may fail to generate stable achievement-related feedback, thereby weakening their sense of accomplishment and professional value. At the same time, grassroots sports school coaches simultaneously assume multiple roles as trainers, managers, educators, and coordinators of academic learning and athletic training, and this expansion of role boundaries further increases demands on their time, cognitive capacity, and emotional resources. When such sustained investment is not matched by adequate organizational support, career development opportunities, and psychological resource replenishment, coaches may become more vulnerable to emotional exhaustion and a diminished sense of professional meaning (Yu and Cheng, 2024). Taken together, the findings suggest that reduced occupational wellbeing among sports school coaches may stem from a persistent mismatch among high job demands, limited control over outcomes, multiple role burdens, and insufficient resource compensation.

### 5.2 The mediating role of emotional intelligence

The present study found that emotional intelligence partially mediated the relationship between work pressure and occupational wellbeing among sports school coaches, thereby supporting Hypothesis 2 and broadly aligning with previous findings (Dogru, 2022). This result suggests that the association between work pressure and occupational wellbeing reflects not only the direct psychological strain caused by high job demands, but also coaches' ability to process emotional information in stressful situations. Higher emotional intelligence can help coaches recognize changes in their own emotions in a timely manner and reduce the perceived threat of stressful events through cognitive reappraisal and emotion regulation, thereby alleviating negative experiences such as anxiety, frustration, and emotional exhaustion (Côté, 2014; Mikolajczak et al., 2007). This is particularly important for coaches in grassroots sports schools, as pressures arising from competition outcomes, fluctuations in athletes' performance, and organizational evaluations are often immediate in nature, and effective emotion regulation can help coaches maintain a stable coaching state and positive occupational experiences. Emotional intelligence can therefore be regarded as an important psychological resource in the relationship between working pressure and occupational wellbeing. However, the present study identified only a partial mediating effect, indicating that although emotional intelligence may mitigate the adverse effects of work pressure, it cannot fully offset the resource depletion caused by objective working conditions such as excessive workload, limited job control, and insufficient organizational support. These findings suggest that improving the occupational wellbeing of sports school coaches requires both strengthening individual psychological resources, such as emotion regulation, and improving the external work environment.

### 5.3 The mediating role of self-efficacy

Self-efficacy partially mediated the relationship between work pressure and occupational wellbeing among sports school coaches, supporting Hypothesis 3. This finding is broadly consistent with previous research (Zhan, 2016). Coaches with higher self-efficacy are generally better able to mobilize their professional experience, training knowledge, and psychological resources when confronted with intensive training demands, performance pressure, athlete management problems, and organizational evaluation requirements. They are also more likely to seek support from colleagues, administrators, or professional teams, thereby enhancing their sense of control over coaching work and reducing the negative effects of stress. Previous research has shown that coaching efficacy can predict coaching behaviors, team satisfaction, and team performance, with its influence further reflected in training management and overall team functioning (Myers N. D. et al., 2005; Myers S. et al., 2005). According to Bandura's social cognitive theory, self-efficacy directly influences individuals' cognitive appraisals and behavioral choices when they encounter stressful events, coaching challenges, and complex training situations. Related studies have also suggested that self-efficacy, as an important psychological resource for coaches, can not only mitigate the adverse effects of work pressure on psychological states and occupational experiences, but also enhance personal initiative (Du and Liu, 2016)and alleviate burnout (Wang, 2021). In the context of sports school coaching, self-efficacy reflects not only coaches' confidence in specialized training, technical and tactical instruction, and competition management, but also their perceived ability to deal with athletes' emotional fluctuations, training fatigue, team conflicts, and school-family communication. When coaches believe that they can effectively address practical problems arising in training and management, they are more willing to integrate their experience, professional knowledge, and psychological resources and to seek organizational support, peer collaboration, and professional assistance in coping with coaching challenges. This increased confidence in coaching may encourage coaches to respond more proactively to work-related difficulties, reduce feelings of helplessness, frustration, and burnout, and ultimately enhance occupational wellbeing. Therefore, sports school coaches with higher self-efficacy may not only cope more effectively with work pressure, but also derive a stronger sense of occupational control and achievement from this positive psychological resource, thereby promoting occupational wellbeing.

### 5.4 The chain mediation effect of emotional intelligence and self-efficacy in the relationship between working pressure and occupational wellbeing of sports school coaches

The present study found a significant chain mediating effect of emotional intelligence and self-efficacy in the relationship between work pressure and occupational wellbeing among sports school coaches, thereby supporting Hypothesis 4. This finding suggests that work pressure is associated not only with lower occupational wellbeing directly, but also with lower emotional intelligence, which may in turn be related to reduced self-efficacy and ultimately lower occupational wellbeing. This result is consistent with the core proposition of the Job Demands-Resources (JD-R) model, which emphasizes that whether individuals can maintain positive occupational functioning under high job demands depends largely on their access to and effective use of sufficient job and personal resources. Within the JD-R framework, emotional intelligence may function as an important psychological resource for sports school coaches and may further strengthen their capacity to cope with working pressure by enhancing self-efficacy, another key personal resource (Bakker and Demerouti, 2017). The development of self-efficacy is also closely related to individuals' self-awareness, self-regulation, and emotion-regulation abilities, while judgments of personal competence influence goal selection, behavioral persistence, and coping responses to stress (Bandura, 1999). Accordingly, grassroots sports school coaches with higher emotional intelligence may be more likely to interpret pressures arising from training workload, competition demands, fluctuations in athletes' performance, and organizational evaluations as manageable situations that can be addressed through professional strategies, thereby maintaining stronger coaching confidence and self-efficacy (Iancheva and Prodanov, 2019). This enhanced sense of efficacy may enable coaches to exercise greater control in training and team management and to better support athletes' competitive development and psychological growth through positive coach-athlete interactions, supportive training environments, and individualized training arrangements, thereby generating a stronger sense of professional accomplishment (Chan and Mallett, 2011). Through this positive process of cognitive appraisal and emotion regulation, coaches may further strengthen their self-efficacy and derive greater achievement and success from athlete progress, team development, and training feedback, ultimately contributing to higher occupational wellbeing.

## 6 Conclusions and practical implications

This study indicates that working pressure is negatively correlated with occupational wellbeing, emotional intelligence, and self-efficacy among sports school coaches. Coaches experiencing higher levels of working pressure tend to exhibit lower emotional intelligence and self-efficacy, which in turn undermines their positive occupational experiences. Meanwhile, the results also show that both emotional intelligence and self-efficacy are positively correlated with occupational wellbeing among sports school coaches. This suggests that coaches with stronger emotional understanding, emotional regulation abilities, and higher coaching efficacy beliefs are more likely to maintain positive occupational experiences even in high-stress environments. In addition, it is noteworthy that emotional intelligence and self-efficacy not only function as independent mediators between working pressure and occupational wellbeing, but also form a sequential chain mediation effect.

This study has several practical implications. First, it highlights the importance of working pressure management in the professional development of sports school coaches. Greater attention should be paid to coaches' workload, time pressure, and organizational support. Unnecessary working pressure can be reduced by optimizing performance evaluation systems, minimizing non-training administrative tasks, and improving resource allocation and support mechanisms. Second, the findings provide insights into the psychological mechanisms underlying coaches' experiences, offering useful perspectives and strategies for promoting their professional development. Third, this study contributes to understanding coaches working in grassroots sports institutions by helping to identify and address declines in occupational wellbeing caused by changes in emotional intelligence and self-efficacy under conditions of working pressure. Fourth, the results suggest that the joint enhancement of emotional intelligence and self-efficacy may help alleviate the negative effects of working pressure on coaches' occupational wellbeing, thereby supporting the sustainable development of youth sports talent development in grassroots sports systems.

This study extends the theoretical framework regarding the relationship between working pressure and occupational wellbeing among sports school coaches. The findings indicate that working pressure not only has a direct effect on coaches' occupational wellbeing, but also exerts indirect effects through emotional intelligence and self-efficacy. This further suggests that the occupational wellbeing of grassroots sports school coaches is not solely determined by external working conditions, but is also influenced by their internal psychological resources and stress-coping capacities.

## 7 Limitations and future directions

Although this study systematically examined the relationships among working pressure, emotional intelligence, self-efficacy, and occupational wellbeing among sports school coaches and revealed the chain-mediating roles of emotional intelligence and self-efficacy to some extent, several limitations should be acknowledged. First, potential sampling bias should be considered when interpreting the findings. The initial sample was recruited primarily from Fujian and Zhejiang Provinces, after which snowball sampling through personal referrals was used to expand recruitment to other regions of China. Although this approach facilitated access to a geographically dispersed population of grassroots sports school coaches, it may have resulted in the overrepresentation of coaches who were more accessible through existing interpersonal networks or more willing to participate in the survey. Moreover, sports school coaches across regions may differ in economic development, sports resource allocation, school management models, traditional sport disciplines, and local competitive sports policies. Therefore, the present sample cannot be assumed to be fully representative of all grassroots sports school coaches in China, and the generalizability of the findings to the national population should be interpreted with caution. Second, this study adopted a cross-sectional research design and relied primarily on data collected at a single time point to analyze the relationships among variables. Although the results can reveal associations and mediation pathways among working pressure, emotional intelligence, self-efficacy, and occupational wellbeing, they cannot establish strict causal relationships among these variables. Third, the data were mainly collected through self-report questionnaires completed by coaches. Although measures such as anonymity, non-incentivized participation, and common method bias testing were implemented, self-reported data may still be subject to social desirability bias, subjective judgment, temporary emotional states, and recall bias, which may reduce the objectivity of the findings to some extent. Fourth, this study primarily focused on the chain-mediating roles of emotional intelligence and self-efficacy in the relationship between working pressure and occupational wellbeing. However, the formation of occupational wellbeing among sports school coaches is highly complex and may also be influenced by organizational support, salary and compensation, career development opportunities, leadership style, work engagement, professional identity, psychological resilience, family support, and training resource allocation. These variables were not incorporated into the present model, leaving considerable room for further exploration of the mechanisms underlying coaches' occupational wellbeing. Fifth, the regression-based mediation approach adopted in the present study primarily examines symmetric net effects and average linear relationships among variables and therefore cannot determine whether a particular antecedent condition is necessary for achieving high occupational wellbeing or whether different combinations of conditions may lead to the same outcome.

In response to these limitations, future research could be extended in several directions. First, subsequent studies should employ more rigorous probability-based sampling strategies, such as stratified random sampling or multistage sampling, and recruit coaches from a more balanced range of geographical regions, sport disciplines, professional titles, and levels of coaching experience. Such approaches would reduce potential selection and network-based sampling biases and improve the representativeness and generalizability of the findings. Second, future research could employ longitudinal or follow-up designs to examine changes in working pressure, emotional intelligence, self-efficacy, and occupational wellbeing across different time points, thereby providing stronger evidence regarding temporal ordering and potential causal relationships. Third, future studies could combine multiple data sources, such as interviews, observations, and evaluations from supervisors or colleagues, to reduce the subjective bias associated with single-source self-report questionnaires and enhance the robustness of the findings. Fourth, future research could incorporate additional variables, including organizational support, professional identity, psychological resilience, work engagement, and salary satisfaction, to develop a more comprehensive model of occupational wellbeing among sports school coaches. Fifth, future studies could further validate and extend the proposed model by incorporating Necessary Condition Analysis (NCA) and fuzzy-set Qualitative Comparative Analysis (fsQCA). NCA could identify whether specific antecedent conditions, such as low working pressure, high emotional intelligence, or high self-efficacy, are individually necessary for achieving high occupational wellbeing, thereby complementing the current symmetric findings with an asymmetric, necessity-based perspective. In parallel, fsQCA could identify multiple equifinal configurations of working pressure, emotional intelligence, self-efficacy, and other relevant conditions that jointly contribute to high occupational wellbeing. The combined application of NCA and fsQCA would provide a more comprehensive and complexity-sensitive understanding of how these psychological and occupational resources individually and jointly contribute to coaches' occupational wellbeing.

## Data Availability

The raw data supporting the conclusions of this article will be made available by the authors, without undue reservation.
